# Parasite-Antigen Driven Expansion of IL-5^−^ and IL-5^+^ Th2 Human Subpopulations in Lymphatic Filariasis and Their Differential Dependence on IL-10 and TGFβ

**DOI:** 10.1371/journal.pntd.0002658

**Published:** 2014-01-30

**Authors:** Rajamanickam Anuradha, Parakkal Jovvian George, Luke E. Hanna, Vedachalam Chandrasekaran, P. Paul Kumaran, Thomas B. Nutman, Subash Babu

**Affiliations:** 1 National Institutes of Health—International Center for Excellence in Research, Chennai, India; 2 Laboratory of Parasitic Diseases, National Institutes of Allergy and Infectious Diseases, National Institutes of Health, Bethesda, Maryland, United States of America; 3 National Institute for Research in Tuberculosis, Chennai, India; Leiden University Medical Center, Netherlands

## Abstract

**Background:**

Two different Th2 subsets have been defined recently on the basis of IL-5 expression – an IL-5^+^Th2 subset and an IL-5^−^Th2 subset in the setting of allergy. However, the role of these newly described CD4^+^ T cells subpopulations has not been explored in other contexts.

**Methods:**

To study the role of the Th2 subpopulation in a chronic, tissue invasive parasitic infection (lymphatic filariasis), we examined the frequency of IL-5^+^IL-4^+^IL-13^+^ CD4^+^ T cells and IL-5^−^IL-4 IL-13^+^ CD4^+^ T cells in asymptomatic, infected individuals (INF) and compared them to frequencies (F_o_) in filarial-uninfected (UN) individuals and to those with filarial lymphedema (CP).

**Results:**

INF individuals exhibited a significant increase in the spontaneously expressed and antigen-induced F_o_ of both Th2 subpopulations compared to the UN and CP. Interestingly, there was a positive correlation between the F_o_ of IL-5^+^Th2 cells and the absolute eosinophil and neutrophil counts; in addition there was a positive correlation between the frequency of the CD4^+^IL-5^−^Th2 subpopulation and the levels of parasite antigen – specific IgE and IgG4 in INF individuals. Moreover, blockade of IL-10 and/or TGFβ demonstrated that each of these 2 regulatory cytokines exert opposite effects on the different Th2 subsets. Finally, in those INF individuals cured of infection by anti-filarial therapy, there was a significantly decreased F_o_ of both Th2 subsets.

**Conclusions:**

Our findings suggest that both IL-5^+^ and IL-5^−^Th2 cells play an important role in the regulation of immune responses in filarial infection and that these two Th2 subpopulations may be regulated by different cytokine-receptor mediated processes.

## Author Summary

Th2 cells are CD4^+^ T cells that produce a unique set of cytokines - IL-4, IL-5 and IL-13. Th2 cells are commonly associated with allergies, asthma and helminth infections. A common helminth infection that infects over 120 million people worldwide is lymphatic filarial infection caused by filarial parasites. We show here data that filarial infection is associated with the expansion of two types of Th2 cells, one which produces IL-4 and IL-13 alone without IL-5 and the other which produces all three cytokines. Interestingly, while the former subset is associated with the levels of antibodies - IgG4 and IgE; the latter is associated with the presence of eosinophilia in filarial infected individuals. In addition, these subsets appear to be modulated differently by the immunoregulatory cytokines - IL-10 and TGFβ. Therefore, our study highlights a novel regulation of Th2 cells and suggests that the Th2 compartment is quite heterogeneous in phenotype with possible functional consequences.

## Introduction

Th2 cells were initially characterized as expressing the cytokines – IL-4, IL-5 and IL-13 [1]. Although Th2 cells can express a variety of other cytokines, these three cytokines remain the hallmark Th2 cytokines. Each Th2 cytokine has a well-defined and relatively specific function. While IL-4 is the major driving force behind Th2 differentiation, IgE class switching and alternative macrophage activation, IL-13 is an important mediator of goblet cell hyperplasia, mucus secretion and airway hyper reactivity [2]. IL-5, in contrast, acts primarily on eosinophils and their precursors in the bone marrow to induce enhanced production, survival and activation of these cells [3]. While Th2 cells have generally been considered a homogenous population, recent reports provide evidence for subpopulations within the Th2 lineage [4]. Two of the main subsets identified recently are the IL-5 expressing Th2 subset (co-expressing IL-4, IL-5 and IL-13) and the non IL-5 expressing Th2 subset (co-expressing only IL-4 and IL-13) [4]. Since the three established Th2 cytokines each play a non-redundant role in allergic disease pathology, it was postulated that these Th2 subsets might play an important role in allergic diseases. Indeed, IL-5^+^Th2 cells have been found in greater frequencies (F_o_) in patients with eosinophilic gastrointestinal disease, while peanut allergy was found to be associated with higher F_o_ of IL-5^−^Th2 cells [5].

The canonical host immune response seen in human filarial infections is of the Th2 type and involves the production of cytokines – IL-4, IL-5, IL-9, IL-10 and IL-13, the antibody isotypes – IgG1, IgG4 and IgE, and expanded populations of eosinophils and immunoregulatory monocyte [6]. Human filarial infection is known to be associated with down regulation of parasite-specific Th1 responses and T cell proliferation and but with augmented Th2 responses [7]. Thus, in human lymphatic filariasis (LF) patent filarial infection is associated with an antigen – specific expansion of Th2 cells (mostly defined by IL-4 expression) and enhanced production of IL-4 and IL-13 [7]. However, antigen – driven IL-5 production has been shown to be diminished in patently infected individuals [8], [9] in some studies. Similarly, although protective immunity to filarial infections in mice is dependent primarily on IL-4, IL-5 does not appear to play a role in resistance to infection [10], [11]. Hence, filarial infections provide a natural setting in which to explore the differential role (if any) of Th2 subsets. We wanted to explore the hypothesis that Th2 subsets would be differentially regulated in asymptomatic infection compared to uninfected or individuals with chronic pathology.

We, therefore, examined the Th2 cytokine expression patterns of CD4^+^ T cells in clinically asymptomatic patently infected (INF) individuals, filarial-uninfected endemic normal (UN) individuals, and in previously infected patients with filarial lymphedema (CP) both at homeostasis and following stimulation with parasite and control antigens. We show that active human LF is characterized by a significant enhancement in the F_o_ of both spontaneously-expressed and parasite antigen – driven IL-5^−^ and IL-5^+^Th2 cells. We show that the F_o_ of IL-5^+^Th2 subpopulation is positively correlated with peripheral eosinophil and neutrophil numbers in filarial infections, while the IL-5^−^Th2 cells are strongly positively related to the levels of parasite specific IgE and IgG4. We also show that these Th2 subpopulations appear to have differing programs of regulation by both IL-10 and TGFβ in filarial-infected individuals and that definitive treatment (and subsequent cure) of this infection causes reversion of the CD4^+^ Th2 subpopulation expansion to normal levels.

## Methods

### Ethics statement

All individuals were examined as part of natural history studies approved by Institutional Review Boards of both the National Institutes of Allergy and Infectious Diseases and the National Institute for Research in Tuberculosis (NCT00375583 and NCT00001230), and informed written consent was obtained from all participants.

### Study population

The study took place in Tamil Nadu, South India where from 1000 subjects in the area, 70 subjects who were willing to provide blood were included. These subjects originate from hospital and surroundings and therefore may represent very different populations. The 70 individuals comprised of 32 clinically asymptomatic, infected (hereafter INF) individuals, 23 individuals with filarial lymphedema or elephantiasis (hereafter CP) and 15 uninfected, endemic normal (hereafter UN) individuals (Table 1). This was primarily a retrospective study using previously collected samples that had been fixed and cryopreserved. The samples obtained post-treatment were collected prospectively.

**Table 1:** Characteristics of the study population.

	CP^a^	INF	UN
	(n = 23)	(n = 32)	(n = 15)
Median age (range)	42 (29–65)	39 (23–65)	36 (24–65)
Gender male/female	15/8	22/10	9/6
Lymphedema/Elephantiasis	23	0	0
ICT card test	Negative	Positive	Negative
*W. bancrofti* circulating antigen levels (U/ml)	<32^b^	1409 (138–22377)	<32
Hb gm/dL GM (Range)	12.58 (6.9–17.2)	13.49 (10.1–16)	14.39 (10.6–23.6)
RBC 10^6^/µL GM (Range)	4.5 (2.94–5.59)	4.68 (3.71–5.47)	5.02 (3.9–8.39)
WBC 10^3^/µL GM (Range)	7.06 (4.2–11.2)	7.45 (4–10.6)	6.77 (4.5–9.8)
HCT % GM (Range)	36.44 (23–45.6)	40.10 (30.7–46.7)	43.51 (32.2–71.1)
PLT 10^3^/µL	286.39 (171–742)	264.06 (124–378)	236.05 (72–352)
Neutrophil 10^3^/µL GM (Range)	3.93 (2.15–8.17)	3.42 (1.92–6.14)	3.89 (2.42–5.99)
Lymphocyte 10^3^/µL GM (Range)	2.13(1.52–3.49)	2.3 (1.0–4.6)	2.02 (1.17–3.0)
Monocyte 10^3^/µL GM (Range)	0.47 (0.11–0.87)	0.77 (0.27–2.32)	0.324 (0.18–0.47)
Eosinophil 10^3^/µL GM (Range)	0.30 (0.05–0.68)	0.53 (0.15–1.22)	0.27 (0.10–0.59)
Basophil 10^3^/µL GM (Range)	0.03 (0.005–0.09)	0.02 (0.005–0.08)	0.04 (0.02–0.36)

a CP refers to individuals with filarial pathology, INF refers to individuals with asymptomatic, filarial infection and UN refers to uninfected individuals.

b Below the limits of detection.

The study individuals were recruited from individuals attending the Filariasis Clinic of the Government General Hospital, Chennai and from community screening of the areas where the individuals reside (Puliayonthope and Ponneri areas). All CP individuals were circulating filarial antigen negative by both the ICT filarial antigen test (Binax, Portland, ME) and the TropBio Og4C3 enzyme-linked immunosorbent assay (ELISA) (Trop Bio Pty. Ltd, Townsville, Queensland, Australia), indicating a lack of current active infection. The diagnosis of prior filarial infection was made by history and clinical examination as well as by positive *Brugia malayi* antigen (BmA) -specific IgG4. BmA-specific IgE, IgG4 and IgG ELISA were performed exactly as described previously [12]. All INF individuals tested positive for active infection by both the ICT filarial antigen test and the TropBio Og4C3 ELISA and had not received any anti-filarial treatment prior to this study. All INF individuals were treated with a standard dose of diethylcarbamazine (DEC) and albendazole and follow – up blood draws were obtained one year later from 16 individuals. Among the 32 INF individuals, 25 were used for whole blood culture with parasite antigens and 7 were used for cytokine blocking studies. Among the 32 treated individuals, we were able to follow up only 16 individuals out of which 9 were circulating antigen negative (Cured) and 7 remained circulating antigen positive (Not-cured). These individuals were used for post-treatment analysis. We also used 7 of the 32 INF individuals exclusively for performing cytokine blocking studies. All UN individuals were circulating filarial antigen negative and without any signs or symptoms of infection or disease. We have selected individuals from the same community and socio-economic backgrounds to account for exposure and socio-economic status.

### Parasite and control antigen

Saline extracts of *B. malayi* adult worms (BmA) and microfilariae (Mf) were used for parasite antigens and mycobacterial PPD (Serum Statens Institute, Copenhagen, Denmark) was used as the control antigen. Final concentrations were 10 µg/ml for BmA, Mf and PPD. Endotoxin levels in the BmA was <0.1 EU/ml using the QCL-1000 Chromogenic LAL test kit (BioWhittaker). Phorbol myristoyl acetate (PMA) and ionomycin at concentrations of 12.5 ng/ml and 125 ng/ml (respectively), were used as the positive control stimuli.

### Hematological parameters

Leukocyte counts and differentials were performed on all individuals using the Act-5 Diff hematology analyzer (Beckman Coulter). The INF individuals had higher eosinophil counts but did not differ from the other two groups in any of the other hematological parameters (data not shown).

### In vitro culture

Whole blood cell cultures were performed to determine the **F_o_** of intracellular cytokine-producing cells.. Briefly, whole blood was diluted 1∶1 with RPMI-1640 medium, supplemented with penicillin/streptomycin (100 U/100 mg/ml), L-glutamine (2 mM), and HEPES (10 mM) (all from Invitrogen, San Diego, CA) and placed in 12-well tissue culture plates (Costar, Corning Inc., NY, USA). The cultures were then stimulated with BmA, Mf, PPD, PMA/ionomycin (P/I) or media alone in the presence of the co-stimulatory reagent, CD49d/CD28 (BD Biosciences) at 37°C for 6 hrs. FastImmune Brefeldin A Solution (10 µg/ml) (BD Biosciences) was added after 2 hours. After 6 hours, whole blood was centrifuged, washed using cold PBS, and then 1× FACS lysing solution (BD Biosciences, San Diego, CA, USA) was added. The cells were fixed using cytofix/cytoperm buffer (BD Biosciences, San Diego, CA, USA), cryopreserved, and stored at −80°C until use. The same procedure was used for both prospectively collected as well as for retrospectively stored samples. For cytokine neutralization experiments (n = 7), whole blood from INF individuals was cultured in the presence of anti-IL-10 (5 µg/ml) or anti-TGFβ (5 µg/ml) or isotype control antibody (5 µg/ml) (R& D Sytems) for 1 h following which BmA and brefeldin A was added and cultured for a further 5 h.

### Intracellular cytokine staining

The cells were thawed and washed with PBS first and PBS/1% BSA later and then stained with surface antibodies for 30–60 minutes. Surface antibodies used were CD3 - Amcyan, CD4 - APC-H7 and CD8 - PE-Cy7 (all from BD Biosciences). The cells were washed and permeabilized with BD Perm/Wash™ buffer (BD Biosciences) and stained with intracellular cytokines for an additional 30 min before washing and acquisition. Cytokine antibodies used were IL-4 FITC, IL-5 APC and IL-13 PE (all from BD Pharmingen. Flow cytometry was performed on a FACS Canto II flow cytometer with FACSDiva software v.6 (Becton Dickinson). The lymphocyte gating was set by forward and side scatter and 100,000 gated lymphocyte events were acquired. Data were collected and analyzed using Flow Jo software. All data are depicted as F_o_ of CD4^+^ T cells expressing cytokine(s). Frequencies following media stimulation are depicted as baseline F_o_ while F_o_ following stimulation with antigens or PMA/ionomycin are depicted as net F_o_ (with baseline F_o_ subtracted).

### Statistical analysis

Data analyses were performed using GraphPad PRISM v6.0 (GraphPad Software, Inc., San Diego, CA, USA). Geometric means (GM) were used for measurements of central tendency. Comparisons were made using either the Kruskal-Wallis test with Dunn's multiple comparisons (unpaired comparisons) or the Wilcoxon signed rank test (paired comparisons). Correlations were calculated using the Spearman rank test.

## Results

### Increased frequency of IL-5^−^ and IL-5^+^Th2 subsets following filarial – antigen stimulation in INF compared to UN and CP individuals

**Figure 1:**
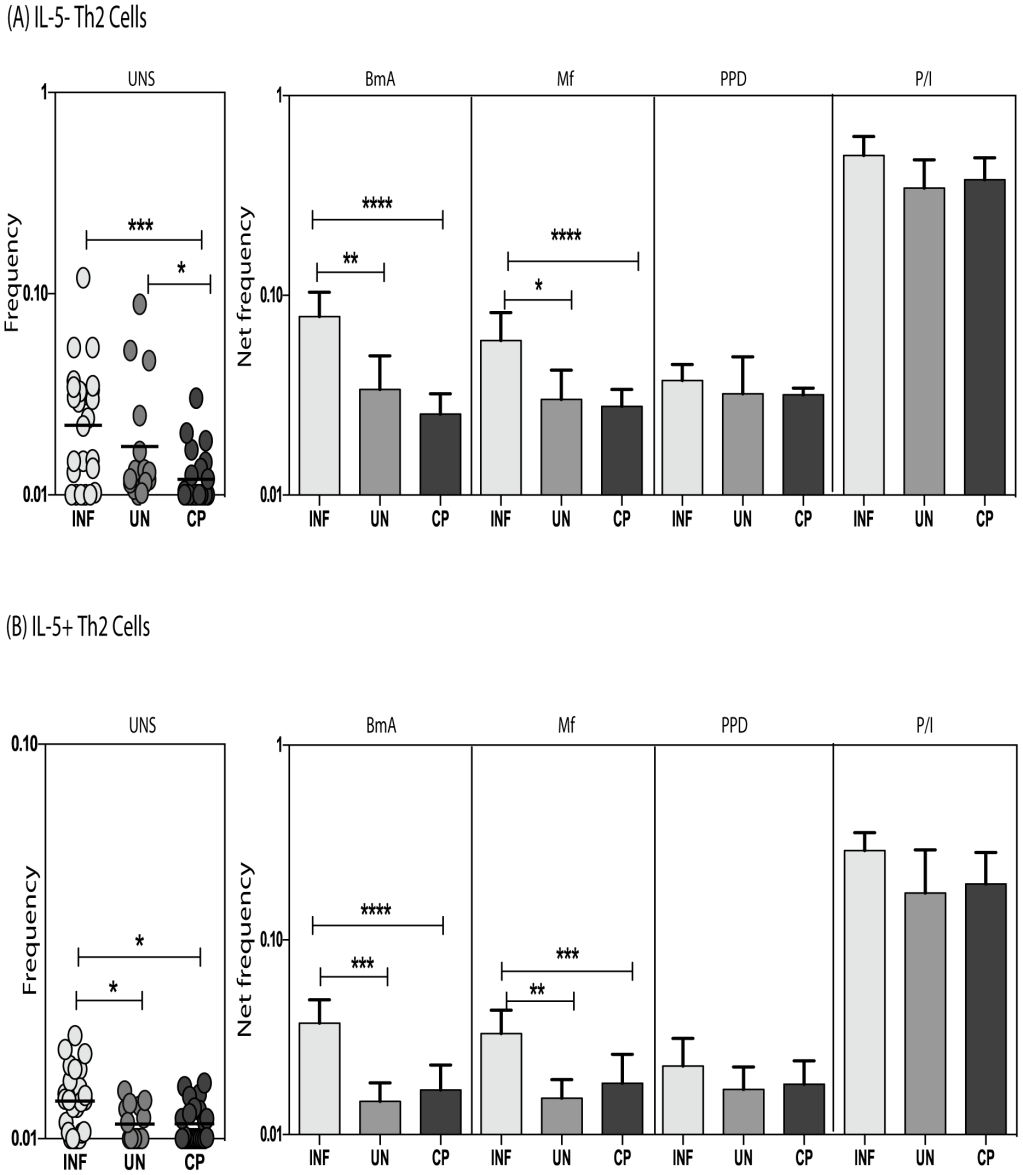
Expanded F_o_ of Th2 subsets in filarial infected individuals in comparison to uninfected individuals and individuals with chronic pathology. (A) Baseline as well as filarial antigen (BmA and Mf), PPD and PMA/ionomycin stimulated F_o_ of CD4^+^ T cells co-expressing IL-4 and IL-13 but not IL-5 (IL-5^−^Th2 cells) in INF (n = 25), UN (n = 15) and CP (n = 23) individuals. (B) Baseline as well as filarial antigen (BmA and Mf), PPD and PMA/ionomycin stimulated F_o_ of CD4^+^ T cells co-expressing IL-4, IL-5 and IL-13 (IL-5^+^Th2 cells) in INF, UN and CP individuals. The data are depicted as bar graphs with the bars representing the geometric means and 95% confidence intervals. P values were calculated using the Kruskal-Wallis test with Dunn's multiple comparisons (* p<0.05, ** p<0.01, *** p<0.001).

We first measured the spontaneously expressed and antigen - stimulated F_o_ of CD4^+^ T cells expressing IL-4 and IL-13 but not IL-5 (CD4^+^IL4^+^IL13^+^IL5^−^) as well as those expressing IL-4, IL-13, and IL-5 (CD4^+^IL4^+^IL13^+^IL5^+^) in INF (for representative flow plot, see Fig. S1) and contrasted these with the F_o_ of these subpopulations in UN and CP. As shown in Figure 1A, INF individuals exhibit significantly higher F_o_ of IL-5^−^Th2 cells in response to the parasite antigens BmA (2.2 fold) and Mf (2.1 fold) but not at baseline nor following PPD or PMA/ionomycin stimulation in comparison to UN individuals, INF individuals also had significantly higher F_o_ of IL-5^+^Th2 cells at baseline (1.2 fold) and following BmA (2.6 fold) and Mf (1.9 fold) (but not PPD or PMA/ionomcyin) stimulation in comparison to UN (Figure 1B). Similarly, INF individuals exhibit significantly higher F_o_ of IL-5^−^Th2 cells at baseline (1.8 fold) and in response to BmA (3 fold) and Mf (2.2 fold) but not following PPD or PMA/ionomycin stimulation in comparison to CP individuals (Figure 1A). Finally, INF individuals also had significantly higher F_o_ of IL-5^+^Th2 cells both at baseline (1.2 fold) and following stimulation with BmA (2.1 fold) and Mf (1.7 fold) (but not with PPD or PMA/ionomcyin) in comparison to CP (Figure 1B). Thus, filarial infection in this population was associated with expanded F_o_ of antigen – stimulated Th2 subpopulations, perhaps indicating a role for these cells in filarial infection and in the prevention of overt pathology.

### IL-5^+^Th2 cells exhibit a positive correlation with absolute eosinophil and neutrophil counts in INF individuals

**Figure 2:**
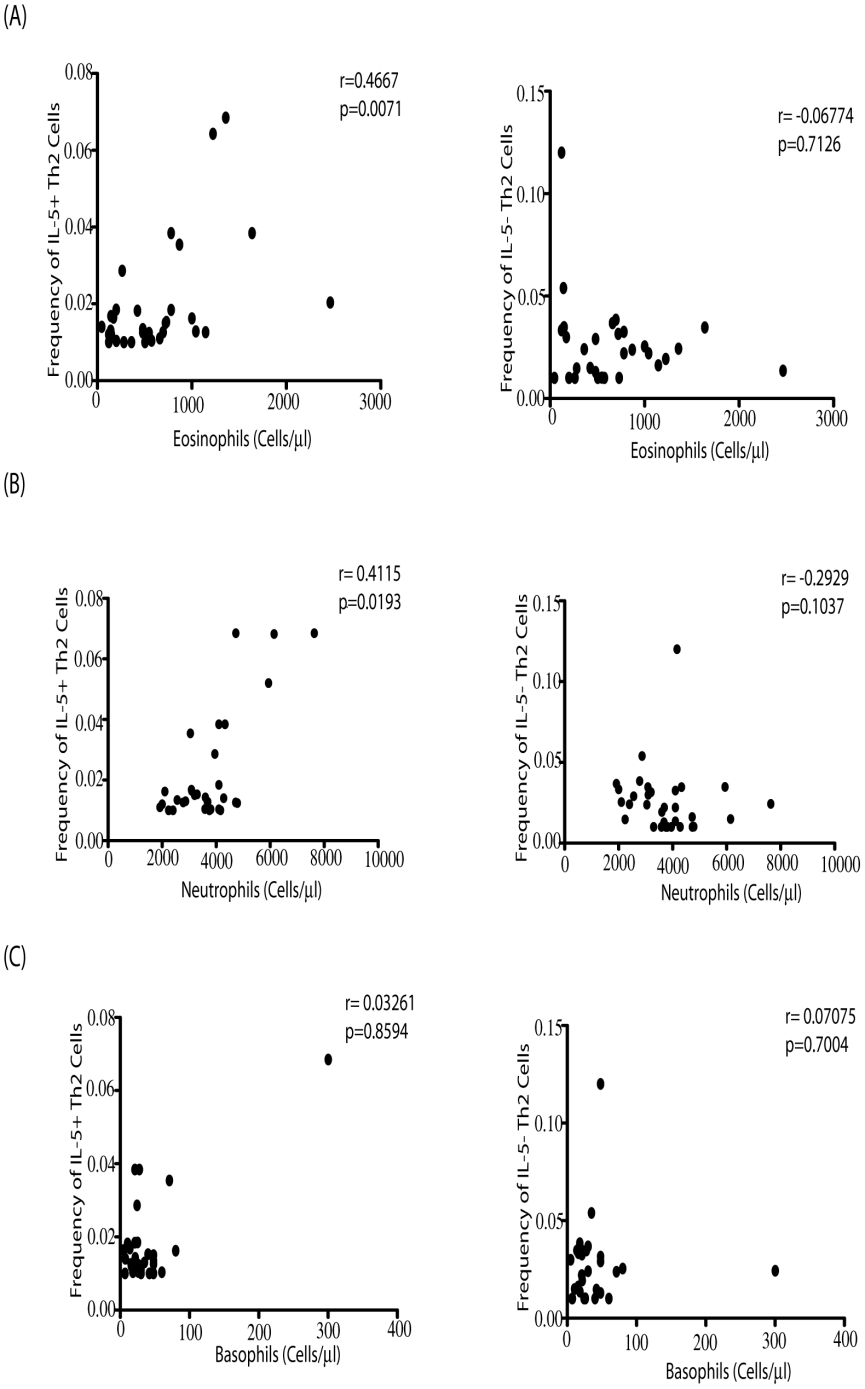
Positive relationship between IL-5^+^Th2 cells and absolute eosinophil and neutrophil counts in filarial infections. Baseline F_o_ of CD4^+^ T cells co-expressing IL-4, IL-5 and IL-13 (IL-5^+^Th2 cells) or co-expressing IL-4 and IL-13 but not IL-5 (IL-5^−^Th2 cells) were correlated to absolute eosinophil (A), neutrophil (B) and basophil (C) counts in INF (n = 32) individuals. The data are shown as scatterplots with each circle representing a single INF individual. P and r values were calculated using the Spearman rank test.

To determine whether the IL-5^−^ and IL-5^+^Th2 subsets were associated with differential functions in filarial infections, we examined the relationship between the baseline F_o_ of IL-5^−^ and IL-5^+^Th2 cells and peripheral eosinophil, neutrophil and basophil numbers in INF individuals (n = 32). As shown in Figure 2A, IL-5^+^ (but not IL-5^−^) Th2 cells had a significantly positive correlation between their F_o_ ex vivo and the absolute eosinophil count (r = 0.4667, p = 0.0071) as determined by Spearman rank correlation. Similarly, as shown in Figure 2B, IL-5^+^ (but not IL-5^−^) Th2 cells exhibited a significant positive association with the absolute neutrophil count (r = 0.4115, p = 0.0193). In contrast, both IL-5^−^ and IL-5^+^Th2 cells showed no correlation with the absolute basophil numbers in INF individuals (Figure 2C).

### IL-5^−^Th2 cells exhibit a positive correlation with IgE and IgG4 isotypes in INF individuals

**Figure 3:**
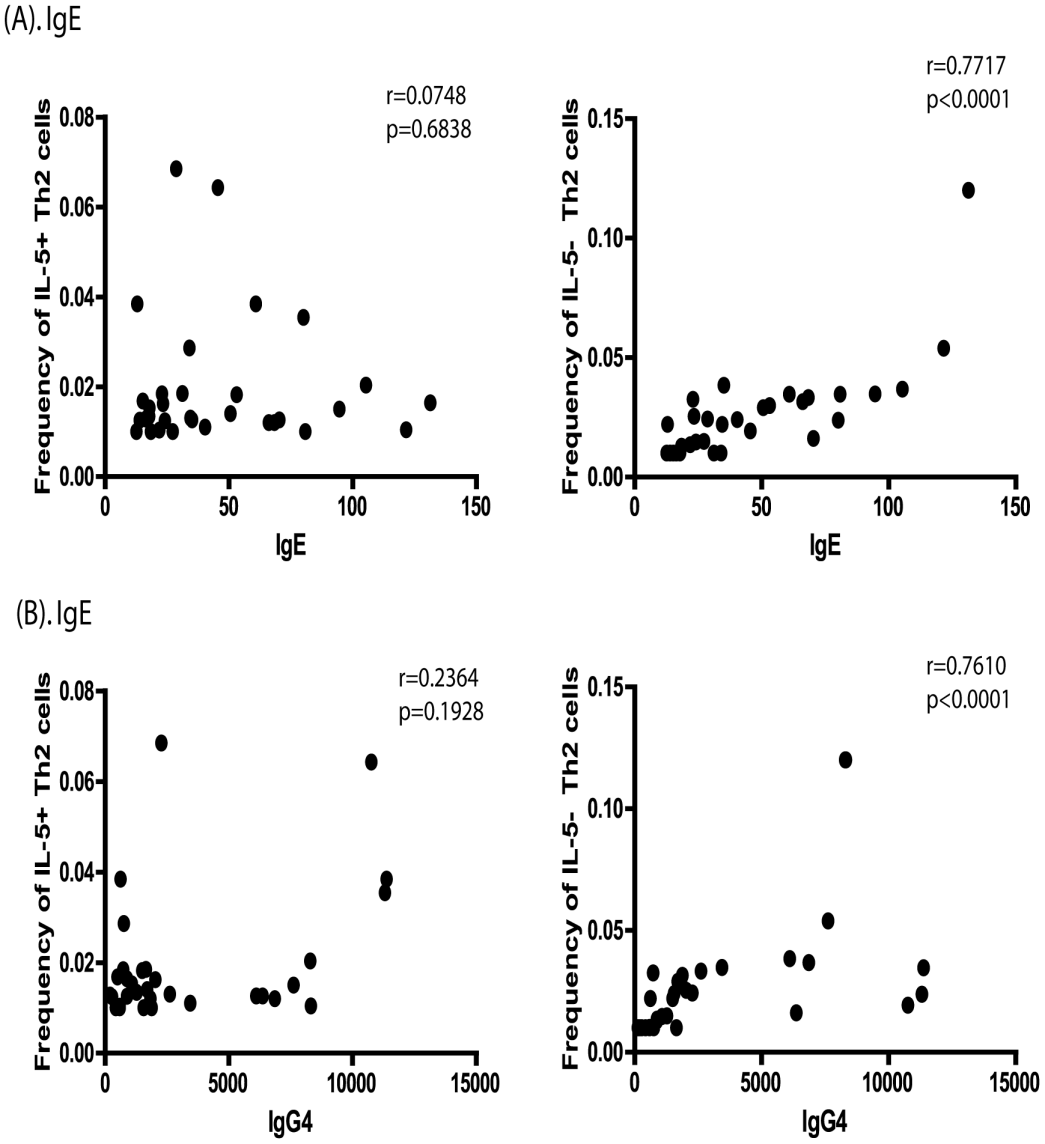
Positive relationship between IL-5^−^Th2 cells and BmA specific IgE and IgG4 levels in filarial infections. Baseline F_o_ of CD4^+^ T cells co-expressing IL-4, IL-5 and IL-13 (IL-5^+^Th2 cells) or co-expressing IL-4 and IL-13 but not IL-5 (IL-5^−^Th2 cells) were correlated to BmA specific IgE (A) or IgG4 (B) antibody titres in INF (n = 32) individuals. The data are shown as scatterplots with each circle representing a single INF individual. P and r values were calculated using the Spearman rank test.

We next examined the relationship between the F_o_ of IL-5^−^ and IL-5^+^Th2 cells ex vivo and IgE and IgG4 levels in INF individuals (n = 32). As shown in Figure 3A, IL-5^−^ (but not IL-5^+^) Th2 cells exhibited a significantly positive correlation between baseline F_o_ and the BmA – specific IgE levels (r = 0.7717, p<0.0001) as determined by Spearman rank correlation. Similarly, as shown in Figure 3B, IL-5^−^ (but not IL-5^+^) Th2 cells exhibited a significant positive association with the BmA – specific IgG4 levels (r = 0.4115, p = 0.0193).

### Th2 subsets are differentially regulated by IL-10 and TGFβ in INF individuals

**Figure 4:**
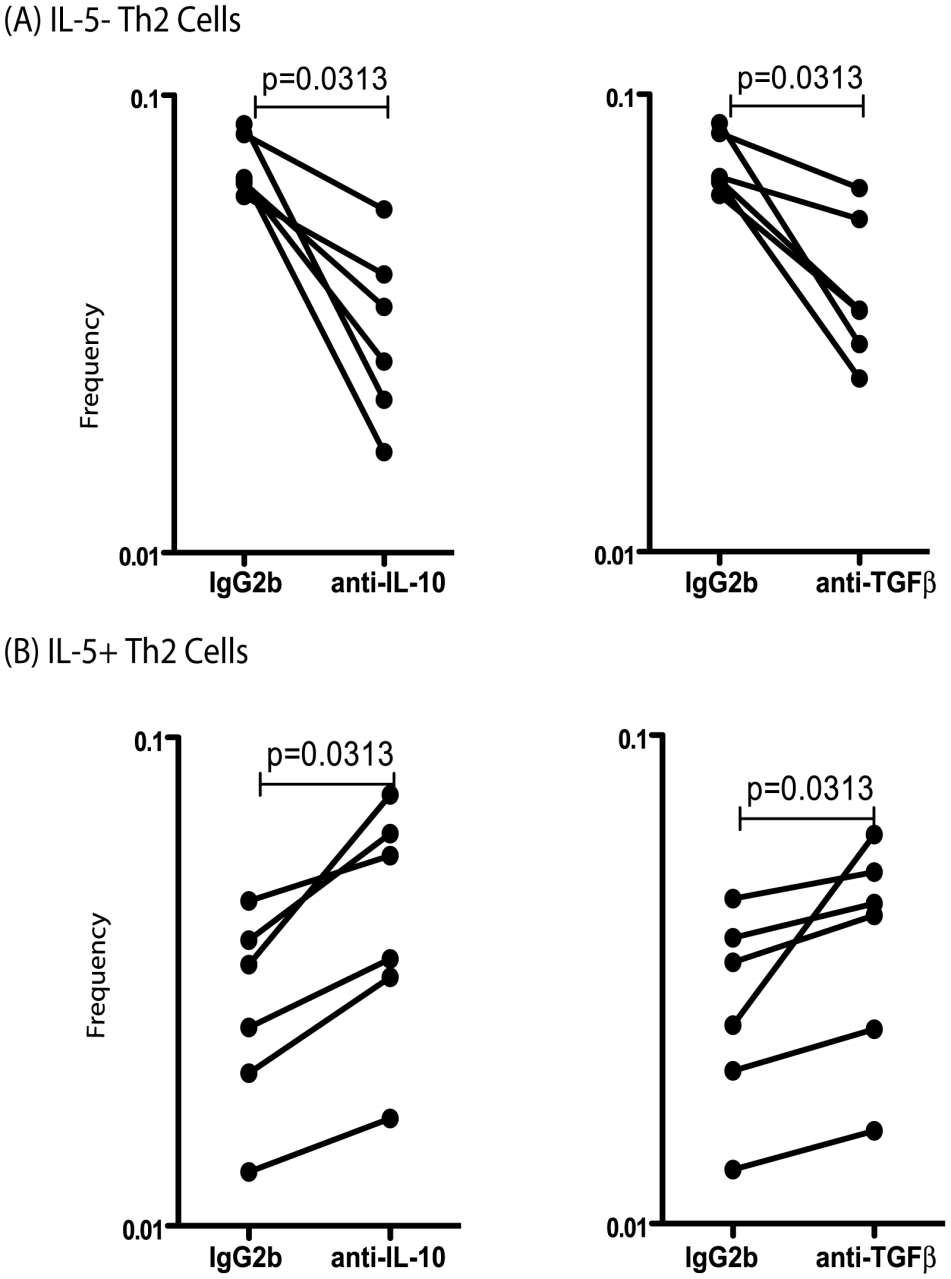
IL-10 and TGFβ regulate the F_o_ of Th2 subsets in filarial infections. (A) IL-10 and TGFβ neutralization (with anti-IL-10 and anti- TGFβ antibodies, respectively) significantly decreases the F_o_ of CD4^+^ T cells expressing IL-4 and IL-13 but not IL-5 (IL-5^−^Th2 cells) following stimulation with BmA in a subset of INF individuals (n = 7). (B) IL-10 and TGFβ neutralization significantly increases the F_o_ of CD4^+^ T cells expressing IL-4, IL-5 and IL-13 (IL-5^+^Th2 cells) following stimulation with BmA in a subset of INF individuals. Antigen – stimulated F_o_ are shown as net F_o_ with the baseline levels subtracted. Each line represents a single individual. P values were calculated using the Wilcoxon signed rank test.

To determine the role of IL-10 and TGFβ in the modulation of Th2 subpopulations in INF, we measured the frequency of IL-5^−^Th2 cells and IL-5^+^Th2 cells following short-term stimulation with the parasite antigen BmA in the presence or absence of anti-IL-10 or anti-TGFβ neutralizing antibody in INF individuals (n = 7). As shown in Figure 4A, both IL-10 and TGFβ neutralization resulted in significantly decreased F_o_ of IL-5^−^Th2 cells in INF individuals (2. 3 and 1.9 fold respectively). In marked contrast, as shown in Figure 4B, both IL-10 and TGFβ blockade resulted in significantly increased F_o_ of IL-5^+^Th2 cells following BmA stimulation (1.5 and 1.3 fold respectively). Thus, both IL-10 and TGFβ play an important role in the modulation of Th2 subset F_o_ in filarial infections.

### Treatment of filarial infection decreases the frequency of antigen – stimulated Th2 subsets in INF individuals

**Figure 5:**
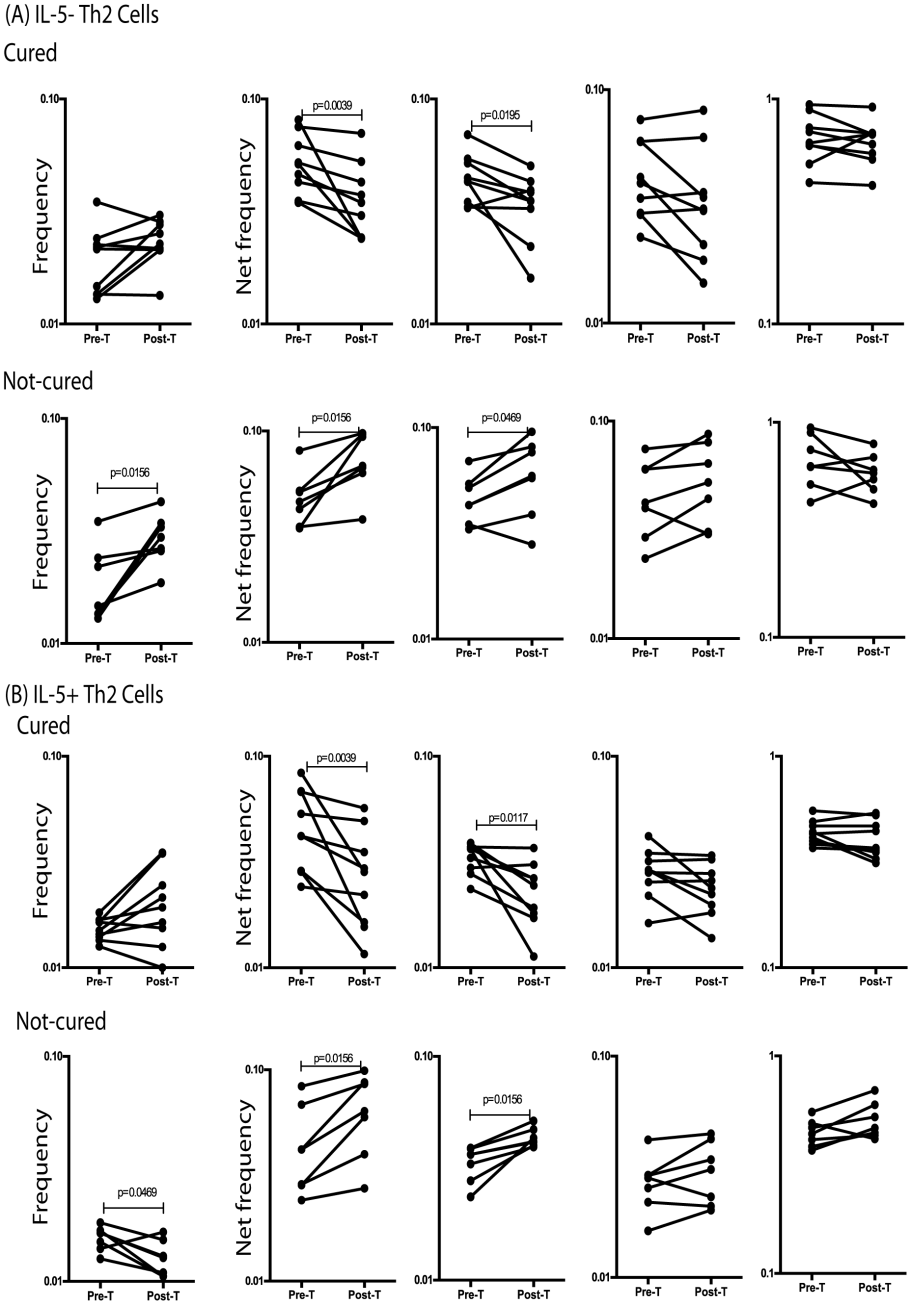
Treatment of filarial infection is associated with decreased F_o_ of antigen – specific Th2 subsets. (A) The F_o_ of IL-5-Th2 cells at baseline and following stimulation with BmA, Mf, PPD and PMA/ionomycin before and after treatment with a standard dose of DEC and albendazole in a subset of INF individuals (n = 16), who were either cured (n = 9) or not cured (n = 7). (B) The F_o_ of IL-5^+^Th2 cells at baseline and following stimulation with BmA, Mf, PPD and PMA/ionomycin before and after treatment with a standard dose of DEC and albendazole in a subset of INF individuals (n = 16), who were either cured (n = 9) or not cured (n = 7). Antigen – stimulated F_o_ are shown as net F_o_ with the baseline levels subtracted. Each line represents a single individual. P values were calculated using the Wilcoxon signed rank test.

To determine the role of antigen – persistence in the regulation of Th2 subsets in filarial infections, we measured the frequency of IL-5^−^Th2 and IL-5^+^Th2 cells in a subset of INF individuals (n = 9), who had been treated with anti-filarial chemotherapy and as a result had eliminated infection (as demonstrated by the absence of circulating filarial antigen) or those who had been treated but continued to harbor infection (n = 7). As shown in Figure 5A, treatment of filarial infection and consequent cure resulted in significantly decreased F_o_ of IL-5^−^Th2 cells upon filarial antigen stimulation (1.4 fold for BmA and 1.3 fold for Mf) when compared to their pre-treatment levels. Similarly, treatment also resulted in significantly decreased F_o_ of IL-5^+^Th2 cells following filarial antigen stimulation (1.7 fold for BmA and 1.5 fold Mf) (Figure 5B). However, individuals who were treated but did not eliminate infection continued to exhibit significantly increased F_o_ of IL-5^−^ and IL-5^+^Th2 cells in response to filarial antigens compared to pre-treatment F_o_ (Figure 5). Interestingly, this effect was specific to the filarial – antigen stimulated F_o_ of CD4^+^ Th2 subpopulations as neither responses to PPD nor to PMA/ionomycin were significantly among the groups. Thus, the antigen – driven expansion of Th2 subpopulations appears to be dependent on the continued presence of circulating filarial antigen (an indicator of active infection).

## Discussion

Th2 responses are considered to be the hallmark of helminth infection and are indeed required for host resistance to a variety of helminths in animals [6]. The three prototypical Th2 cytokines – IL-4, IL-5 and IL-13 have all been shown to play important but non – redundant roles in helminth immunity [13]. In addition, the recent explosion of interest in CD4^+^ T cell subpopulations and the availability of polychromatic cytokine staining has helped define heterogeneity within the Th2 compartment [4]. Thus, two major subsets of Th2 cells were recently described – an IL-5 expressing Th2 population, which is thought to play an important role in eosinophilic inflammation and an IL-5 non expressing Th2 population, which is thought to play an important role in other forms of allergic inflammation [4]. Moreover, it has been demonstrated that IL-5^+^ and IL-5^−^Th2 cells represent more and less highly differentiated Th2 subpopulation, respectively [14]. However, the role of these subsets in helminth infections is not known.

The induction of prototypical Th2 response with high IL-4, IL-5 and IL-13 secretion has long been considered to be the hallmark of active infection in human LF [7]. However, not all studies have consistently shown a predominant prototypical Th2 response in filarial infections. A recent study in Mali suggested that patent, long-standing filarial infection is associated with expanded adaptive regulatory T cell cells rather than an expansion of classical Th2 cells environment [15]. Previous studies have reported a down regulation of IL-5 upon parasite stimulation [8], [9], [16]. In addition, the role of Th2 responses in protection from or in the pathogenesis underlying the disease associated with LF has not been well characterized either. We therefore utilized two sets of comparisons to help elucidate the role of Th2 subsets in human filarial infections – (1) comparisons of Th2 subsets in INF and UN individuals, (2) comparisons of these subsets in INF and CP individuals. We were able to demonstrate that both Th2 subsets are expanded preferentially in active, subclinical infection but not in filarial disease (without active infection). Our data on Ag – induced expression of CD4^+^ Th2 cell subpopulations also reveal interesting facets of T cell driven immune regulation in filarial infection and disease. First, the alterations in the CD4^+^ Th2 cell cytokine repertoire is filarial – antigen specific since the since these alterations in Th2 subpopulation F_o_ were primarily observed only in response to the filarial-derived BmA and Mf antigens but not to PPD nor in response to polyclonal stimulation. Second, the importance of antigen – persistence is clearly illustrated by our data on a small subset of individuals who cleared infection following treatment and were therefore proven to be filarial antigen negative. The follow up data on these individuals indicate a clear reversion to the normal/homeostatic levels of Th2 subset populations. On the other hand, the different Th2 subpopulations continue to expand in a control group of individuals, who also received treatment but failed to clear infection. Therefore, the expansion of antigen - specific Th2 subsets is closely associated with the presence of parasite antigen in vivo.

Th2 cells are thought to play a counter-regulatory role in a variety of infectious and inflammatory conditions to offset pathology and promote tissue repair and wound healing mechanisms [17]. Th2 responses are considered to be fundamentally important in protection against the development of pathology both because of their ability to ameliorate Th1 induced inflammatory responses and because of their propensity to promote wound healing and tissue repair [18]. For example, IL-5 and IL-13 have pro-fibrotic activity and, in addition, IL-4 and IL-13 are critical mediators of alternative activation of macrophages. Our study of the Ag – stimulated expansion of Th2 subpopulations reveals a significant association of these cells with asymptomatic infection, confirming a previously reported association [19]. By contrasting these Th2 subpopulations in clinically asymptomatic patients to those with filarial lymphedema (CP) we may be able to infer a role for these expanded Th2 subsets in protection from the development of clinical pathology. Moreover, our data on the lower levels of Th2 subpopulations in CP supports the suggestion that unchecked parasite-specific Th1/Th17 cells may contribute to the pathological process in LF.

Our data reveal clear distinctions in the relationship between IL-5^−^ and IL-5^+^Th2 cells and expansion of innate leukocyte populations in filarial infections. Eosinophils are considered to be important innate effectors in immunity to helminth infections and have been shown to play a role in protection against *S. mansoni* and other helminths [20], [21]. Similarly, basophils are known to act as effectors to promote parasite killing during challenge infections of immunized animals, perhaps through antibody dependent mechanisms [22], [23], while neutrophils have been demonstrated to attack helminth larvae in response to IL-4 and IL-5 [24], [25]. Our study implicates the IL-5^+^Th2 subpopulation in this innate defense mechanism by promoting the recruitment and/or activation of eosinophils and neutrophils. Our study also demonstrates an important association of IL-5^−^Th2 cells in promoting Th2 associated (IgE and IgG4) antibody responses in filarial infection. All helminth infections are characterized by the induction of antibody isotypes of the class – IgE and IgG4 (IgG1 in mice), that are largely dependent on the IL-4 [26].

Not only did we assess the expansion of these Th2 subpopulations, we also examined the mechanisms regulating the expression of these cytokines in these two subpopulations. Since IL-10 and the TGFβ in chronic infections are known to play a role in modulating T cell expression of cytokines in filarial infections [19], we examined the F_o_ of IL-5^+^ and IL-5^−^Th2 cells following either IL-10 or TGFβ blockade during in vitro stimulation with filarial antigen. Our data, through preliminary due to the small number of samples able to obtained, show clear differences in the modulation of the Th2 subsets. We demonstrate that the expansion of IL-5^−^Th2 cells is dependent on both IL-10 and TGFβ since blockade of these cytokines significantly reduces the frequency of IL-5^−^Th2 cells. Conversely, both IL-10 and TGFβ appear to impair the induction of the IL-5^+^Th2 subset. While it has been previously shown that IL-5 expression in Th2 cells is limited to the effector memory subset whereas IL-4 is expressed in both central and effector memory subsets [4], this finding that IL-10 and TGFβ signaling may be critical to Th2 subpopulation expansion provide new insight into the interrelationship between the IL-5^+^ and IL-5^−^Th2 subpopulations and provides new avenues for the study of filarial-specific immune regulation and protection from immune-mediated pathology in LF.

In summary, our study examines in depth the CD4^+^ Th2 cell subset repertoire in a chronic parasitic infection and sheds light on the role of these subsets in both the regulation of immune responses in active infection and in the pathogenesis of filarial lymphedema. While we have not performed longitudinal studies to define the development of pathology in filarial infection and this was a study using a combination of previously (pre-treatment) and prospectively (post-treatment) collected samples with inherent potential limitations with respect to bias, our strategy of contrasting immune responses in individuals with early or subclinical disease and those with late or clinical disease yields important information on the association of Th2 subsets in pathogenesis. However, the potential drawbacks in the study include potential bias in using both prospectively and retrospectively collected samples and lack of rigorous controls in eliminating potential confounders including socio-economic status of individuals in the study. The lack of proper information in the study area regarding the prevalence of the different clinical groups, adds to the problem of potential bias which means that our conclusions cannot be generalized. In addition, while we have demonstrated the presence of Th2 subsets in filarial infections, disease association is not formal proof of function and the elucidation of function needs to be explored in the future. Nevertheless, our study clearly defines and important association of filarial infection with heightened expansion of Th2 cells suggesting that these subsets play an important role in infection.

## Supporting Information

Figure S1 **A representative dot plot showing the BmA – stimulate expression of CD4^+^ T cells expressing various Th2 cytokines.** CD4^+^ T cells expressing IL-4, IL-5 and IL-13 at baseline and following filarial antigen stimulation are shown in a representative flow cytometry plot from an INF individual.
(TIF)
