# Supplementary figures and images for: Parasite-Antigen Driven Expansion of IL-5− and IL-5+ Th2 Human Subpopulations in Lymphatic Filariasis and Their Differential Dependence on IL-10 and TGFβ
Source: PLoS Negl Trop Dis. 2014 Jan 30;8(1):e2658. doi: 10.1371/journal.pntd.0002658 (PMC3907332; doi:10.1371/journal.pntd.0002658)

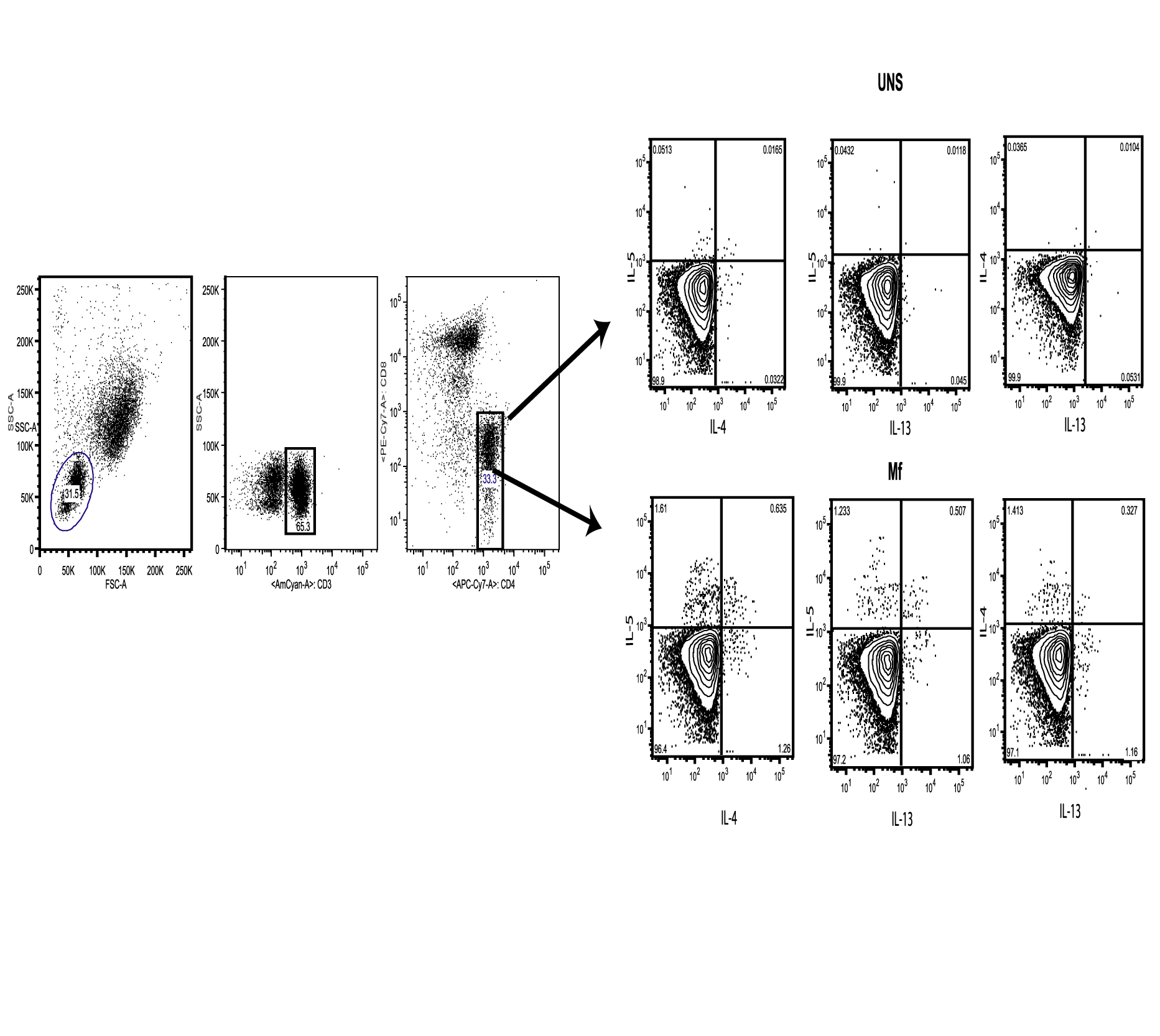

Supplement: Figure S1 — A representative dot plot showing the BmA – stimulate expression of CD4+ T cells expressing various Th2 cytokines. CD4+ T cells expressing IL-4, IL-5 and IL-13 at baseline and following filarial antigen stimulation are shown in a representative flow cytometry plot from an INF individual. (TIF) [file pntd.0002658.s001.tif]
